# HbA1c Variability as a Predictor of Neurocognitive Decline in Children With Type 1 Diabetes

**DOI:** 10.7759/cureus.99141

**Published:** 2025-12-13

**Authors:** Malik Asfand Yar, Falah Hassan Abid, Abdul Manan, Shazia Bahar, Jawaria Majeed, Muhammad Azhar khan, Faisal Akram, Arsallan Siddiqui, Maleeha Rauf, Saad Elahi

**Affiliations:** 1 Acute Medicine, Queen Elizabeth Hospital, University Hospitals Birmingham NHS Foundation Trust, Birmingham, GBR; 2 Pediatrics, Ash-Satrah General Hospital, Dhi-Qar Health Directorate, Dhi-Qar, IRQ; 3 Ear, Nose, and Throat, Worcestershire Acute Hospitals NHS Trust, Worcestershire, GBR; 4 Pediatrics, Khyber Teaching Hospital, Peshawar, PAK; 5 Gastroenterology and Hepatology, Nishtar Medical University and Hospital, Multan, PAK; 6 Community Medicine, Combined Military Hospital (CMH) Kharian Medical College, Kharian, PAK; 7 Family Medicine, Select Medical Clinics, Victoria, AUS; 8 Medicine, Punjab Medical College, Faisalabad, PAK; 9 Neurology, Allied Hospital, Faisalabad, PAK

**Keywords:** children, executive function, hba1c variability, neurocognitive decline, type 1 diabetes

## Abstract

Background: Children with type 1 diabetes mellitus (T1DM) are vulnerable to neurocognitive impairments due to fluctuating glycemic control. Recent evidence suggests that long-term variability in HbA1c, rather than mean HbA1c alone, may be a more sensitive indicator of metabolic instability contributing to cognitive decline.

Objective: This study aimed to evaluate the association between HbA1c variability and neurocognitive performance in children with T1DM and to determine whether HbA1c variability serves as an independent predictor of neurocognitive decline.

Methods: A retrospective analysis was conducted at Khyber Teaching Hospital, Peshawar, Pakistan, including 180 children with T1DM (96 males, 53.3%; 84 females, 46.7%) aged 12.6 ± 3.4 years. Data from the preceding 24 months were reviewed, including serial HbA1c values, clinical characteristics, and standardized neurocognitive test scores. HbA1c variability was quantified using standard deviation (SD) and coefficient of variation (CV). Participants were categorized into high-variability (CV > 8%) and low-variability (CV ≤ 8%) groups. Cognitive domains assessed included full-scale intelligence quotient (IQ), working memory, processing speed, attention, and executive function. Statistical analyses included correlation and multiple linear regression to identify independent predictors of cognitive outcomes.

Results: The mean duration of diabetes was 5.8 ± 2.1 years, and the mean HbA1c was 8.6 ± 1.2%. Children in the high-variability group (n = 89, 49.4%) showed significantly lower full-scale IQ (89.0 ± 10.1 vs. 95.8 ± 9.4, p < 0.001), working memory (85.9 ± 9.1 vs. 93.6 ± 10.3, p = 0.001), processing speed (86.7 ± 10.7 vs. 94.1 ± 11.5, p = 0.003), and executive function (86.5 ± 8.8 vs. 92.9 ± 9.5, p = 0.012) compared with the low-variability group. HbA1c CV showed the strongest negative correlation with full-scale IQ (r = -0.562, p < 0.05). In multivariate regression, HbA1c CV (β = -0.42, p < 0.001) and hypoglycemia frequency (β = -0.21, p = 0.008) independently predicted lower IQ scores.

Conclusion: Higher HbA1c variability is significantly associated with poorer neurocognitive outcomes in children with T1DM, independent of mean HbA1c levels. Long-term glycemic stability may therefore be a crucial target to preserve cognitive function in pediatric diabetes care.

## Introduction

Children with type 1 diabetes (T1DM) are at an increased risk of experiencing deficits in neurocognitive functioning compared to their non-diabetic peers, particularly in domains such as memory, learning, and executive function, which encompass working memory, attention shifting, and response inhibition [1]. Neuroimaging studies have consistently demonstrated structural and developmental differences in the brains of children with T1DM, especially in cerebral white matter regions, suggesting that diabetes may adversely affect brain maturation and connectivity [2,3]. Several clinical and metabolic factors have been proposed to influence the risk or severity of these neurocognitive impairments, including early age of onset [1], presence of diabetic ketoacidosis (DKA) at diagnosis [4], episodes of severe hypoglycemia [5], and chronic hyperglycemia [6]. However, the evidence remains inconsistent, as prior studies have often been limited by small sample sizes, cross-sectional designs, or inclusion of older adolescents and adults rather than younger pediatric populations.

Clarifying the determinants of neurocognitive dysfunction in children with T1D, particularly problems related to learning, memory, processing speed, and executive function, is essential for refining clinical management and educational support. Earlier recommendations for glycemic targets in children prioritized the avoidance of hypoglycemia due to its suspected association with neuropsychological impairment [4]. Consequently, the 2025 American Diabetes Association (ADA) Standards of Care for Children advised maintaining HbA1c levels between 7.5% and 8.5% for those under six years of age and <8% for those aged six to 12 years, slightly higher than the targets for adolescents and adults (<7.5%) [7]. However, emerging evidence linking chronic hyperglycemia and increased glycemic variability to abnormal brain development and neurocognitive impairment [8] prompted revisions in international guidelines, which now recommend a target HbA1c of <7% for all children and adolescents.

Recent studies have also emphasized the significance of time in range (TIR), the percentage of time glucose levels remain between 70 and 180 mg/dL, as a dynamic marker of glycemic control that correlates with HbA1c and is inversely associated with diabetes-related complications [9]. Among the cognitive domains, executive function appears to be particularly vulnerable to glycemic dysregulation [3]. As children with T1DM gradually assume more responsibility for their diabetes management, impairments in executive functioning may hinder their ability to plan, problem-solve, and adhere to complex treatment regimens [1]. This, in turn, can perpetuate poor glycemic control, creating a feedback loop where metabolic instability further exacerbates neurocognitive decline [10].

Despite the established links between chronic hyperglycemia and cognitive dysfunction, the role of HbA1c variability, reflecting fluctuations in long-term glycemic control, has not been sufficiently explored as a predictor of neurocognitive decline in children with T1DM. Understanding this relationship is crucial, as HbA1c variability may represent a more sensitive marker of glycemic instability and its cumulative impact on the developing brain than mean HbA1c alone. The present study aims to evaluate the association between HbA1c variability and neurocognitive decline in children with T1DM to determine whether fluctuations in glycemic control serve as an independent predictor of cognitive dysfunction in this population.

## Materials and methods

This retrospective observational study was conducted in the Department of Pediatric Endocrinology and Neurology, Khyber Teaching Hospital, Peshawar, Pakistan, over a period of 24 months (from January 2023 to December 2024). The study aimed to investigate the association between HbA1c variability and neurocognitive decline in children diagnosed with T1DM. Ethical approval for the study was obtained from the Institutional Review Board of Khyber Teaching Hospital prior to data collection (approval number: DIR/KMU/-EB/DR/12-36).

The medical records of children aged six to 18 years with a confirmed diagnosis of T1DM for at least two years were reviewed. The sample size was calculated using OpenEpi software (Dean AG, Sullivan KM, & Soe MM, 2013). OpenEpi: Open Source Epidemiologic Statistics for Public Health (Version 3.01). www.OpenEpi.com), assuming an anticipated effect size of 0.3, a confidence level of 95%, and a statistical power of 80%. The sample size calculation followed the standard formula for comparing means:

$n=\frac{2(Z_&alpha;/_2 + Z_&beta;)^2 \sigma^2}{d^2}$

In this formula, n represents the required sample size per group. The value of Zα/2 was set at 1.96, corresponding to a 95% confidence interval (CI), while Zβ was taken as 0.84, representing a statistical power of 80%. The estimated standard deviation (σ) of the outcome variable was assumed to be 1.0, based on previous studies, and the minimum detectable effect size (d) was defined as 0.3 × σ (i.e., 0.3). Thus, a total sample size of 180 participants was determined to ensure adequate statistical power for detecting significant associations between HbA1c variability and neurocognitive outcomes. A non-probability consecutive sampling technique was employed to include all eligible participants who met the inclusion criteria during the study period.

Children were included if they had a documented diagnosis of T1DM based on ADA criteria [7], had at least four HbA1c measurements recorded during the past two years, and had undergone at least one standardized neurocognitive assessment during the same period. Patients with a history of neurological disorders unrelated to diabetes (such as epilepsy, traumatic brain injury, or congenital neurodevelopmental syndromes), psychiatric illnesses, or chronic systemic diseases affecting cognition (such as hypothyroidism or renal failure) were excluded. Records with incomplete HbA1c data or missing neurocognitive assessments were also excluded to ensure data reliability.

For each included patient, data were extracted from hospital records, including demographic characteristics (age, sex, and duration of diabetes), clinical information (insulin regimen, frequency of hypoglycemia or DKA episodes), and serial HbA1c values recorded at regular three-month intervals over the previous 24 months. HbA1c variability was calculated using the standard deviation (SD) and coefficient of variation (CV) of all recorded HbA1c measurements per individual, in addition to the mean HbA1c value. High HbA1c variability was defined as a coefficient of variation greater than 8%, while low variability was defined as less than or equal to 8%.

Neurocognitive performance was assessed using age-appropriate, standardized tools. For children aged six to 12 years, the National Institutes of Health (NIH) Toolbox Cognition Battery (Child Version) [11] was utilized, while for adolescents aged 13 to 18 years, the NIH Toolbox Cognition Battery (Adolescent Version) [12] was employed. Subtests evaluating working memory, processing speed, attention, and executive function were specifically analyzed, as these domains are known to be affected by glycemic instability. Cognitive test results were categorized as normal, mildly impaired, or significantly impaired based on standardized percentile scores. The latest neurocognitive assessment results within the 24-month review period were included for analysis.

To assess potential confounding effects, other relevant variables such as age at diagnosis, total duration of diabetes, frequency of hypoglycemic events, and socioeconomic status were also recorded. The relationship between HbA1c variability and neurocognitive outcomes was analyzed after adjusting for these confounders.

Data were entered and analyzed using IBM SPSS Statistics software, version 26 (IBM Corp., Armonk, NY). Continuous variables were expressed as mean ± standard deviation or median (interquartile range), depending on distribution normality, while categorical variables were presented as frequencies and percentages. Between-group comparisons (high vs. low HbA1c variability) were performed using the independent t-test or Mann-Whitney U test for continuous variables and the chi-square test for categorical variables. Pearson or Spearman correlation coefficients were used to evaluate the association between HbA1c variability indices (SD and CV) and neurocognitive test scores. Multiple linear regression models were constructed to identify independent predictors of neurocognitive decline while controlling for potential confounders such as mean HbA1c, duration of diabetes, and frequency of hypoglycemia. A p-value of less than 0.05 was considered statistically significant.

The study was conducted in accordance with the Declaration of Helsinki, and patient confidentiality was strictly maintained by anonymizing all extracted data. Only authorized research personnel had access to the database, and no direct patient contact occurred due to the retrospective nature of the study.

## Results

A total of 180 children with T1DM were included in the study after screening 213 medical records, of which 33 were excluded due to incomplete HbA1c or neurocognitive data. The final sample comprised 96 males (53.3%) and 84 females (46.7%) with a mean age of 12.6 ± 3.4 years. The mean duration of diabetes was 5.8 ± 2.1 years, and the average number of HbA1c recordings per participant was eight over the 24-month period.

Demographic and clinical characteristics

Table 1 presents the baseline demographic and clinical characteristics of the study participants. The mean HbA1c was 8.6 ± 1.2%, while the mean HbA1c SD was 0.84 ± 0.25, corresponding to a CV of 9.7 ± 2.4%. Based on HbA1c variability, 89 patients (49.4%) were categorized into the high-variability group (CV > 8%), while 91 (50.6%) were classified as low-variability. The two groups were comparable in terms of age, sex distribution, and duration of diabetes. However, the frequency of documented hypoglycemic events was significantly higher among the high-variability group (p = 0.014).

**Table 1:** Baseline demographic and clinical characteristics of the study participants *Significant at p < 0.05; χ²: chi-square; SD: standard deviation; CV: coefficient of variation; DKA: diabetic ketoacidosis

Variable	Total (n=180)	Low Variability (n=91)	High Variability (n=89)	t / χ² (df)	p-value
Age (years, mean ± SD)	12.6 ± 3.4	12.4 ± 3.5	12.8 ± 3.3	t = -0.79	0.432
Sex (male/female)	96/84	48/43	48/41	χ²(1) = 0.01	0.911
Duration of diabetes (years, mean ± SD)	5.8 ± 2.1	5.6 ± 2.0	6.0 ± 2.2	t = -1.23	0.219
Mean HbA1c (%)	8.6 ± 1.2	8.2 ± 1.0	9.0 ± 1.3	t = -4.38	<0.001*
HbA1c SD	0.84 ± 0.25	0.63 ± 0.14	1.05 ± 0.21	t = -13.18	<0.001*
HbA1c CV (%)	9.7 ± 2.4	7.5 ± 1.3	12.0 ± 2.1	t = -10.36	<0.001*
Hypoglycemia episodes (per year, mean ± SD)	4.8 ± 2.9	3.9 ± 2.2	5.7 ± 3.1	t = -2.47	0.014*
DKA episodes (per year, mean ± SD)	1.6 ± 1.1	1.4 ± 0.9	1.8 ± 1.2	t = -1.87	0.062
Socioeconomic status (middle/low)	102/78	55/36	47/42	χ²(1) = 0.77	0.381

Neurocognitive performance among study participants

Neurocognitive function was assessed using standardized age-specific tests. The mean total intelligence quotient (IQ) score across the cohort was 92.4 ± 10.3. Table 2 shows the comparison of neurocognitive test domains between the high and low HbA1c variability groups. Children in the high-variability group demonstrated significantly lower scores in working memory (p = 0.001), processing speed (p = 0.003), and executive function (p = 0.012), while attention scores were also lower but did not reach statistical significance (p = 0.067).

**Table 2:** Comparison of neurocognitive test scores between high and low HbA1c variability groups *Significant at p < 0.05; IQ: intelligence quotient

Cognitive Domain	Low Variability (Mean ± SD)	High Variability (Mean ± SD)	t-value	p-value
Full scale IQ	95.8 ± 9.4	89.0 ± 10.1	t = 4.31	<0.001*
Working memory index	93.6 ± 10.3	85.9 ± 9.1	t = 3.46	0.001*
Processing speed index	94.1 ± 11.5	86.7 ± 10.7	t = 3.03	0.003*
Attention index	91.4 ± 8.9	88.3 ± 9.8	t = 1.84	0.067
Executive function index	92.9 ± 9.5	86.5 ± 8.8	t = 2.56	0.012*

Correlation between HbA1c variability and neurocognitive outcomes

Correlation analysis demonstrated significant negative associations between HbA1c variability indices and neurocognitive test scores. HbA1c CV exhibited stronger correlations with cognitive decline parameters compared to mean HbA1c, indicating that variability plays a more crucial role than chronic hyperglycemia in cognitive performance deterioration (Table 3).

**Table 3:** Correlation between glycemic indices and neurocognitive scores (n=180) *Significant at p < 0.05; SD: standard deviation; CV: coefficient of variation; IQ: intelligence quotient

Variable	Mean HbA1c	HbA1c SD	HbA1c CV
Full scale IQ (r)	-0.341*	-0.478*	-0.562*
Working memory index (r)	-0.282*	-0.421*	-0.509*
Processing speed index (r)	-0.265*	-0.392*	-0.477*
Executive function index (r)	-0.243*	-0.359*	-0.431*

Multivariate regression analysis of predictors of neurocognitive decline

A multiple linear regression analysis was conducted to determine independent predictors of neurocognitive decline while adjusting for age, sex, duration of diabetes, mean HbA1c, frequency of hypoglycemia, and socioeconomic status. HbA1c CV remained a significant independent predictor of lower full-scale IQ scores (β = -0.42, p < 0.001) and reduced working memory index (β = -0.35, p = 0.002) (Table 4).

**Table 4:** Multivariate regression analysis for predictors of full-scale IQ decline *Significant at p < 0.05; β: beta; SD: standard deviation; CV: coefficient of variation; IQ: intelligence quotient

Predictor Variable	β Coefficient	Standard Error	t-value	p-value
Age (years)	-0.12	0.08	-1.51	0.133
Duration of diabetes (years)	-0.19	0.07	-2.71	0.007*
Mean HbA1c (%)	-0.25	0.10	-2.50	0.013*
HbA1c CV (%)	-0.42	0.09	-4.66	<0.001*
Hypoglycemia frequency	-0.21	0.08	-2.68	0.008*
Socioeconomic status (Low)	-0.14	0.06	-2.33	0.021*

## Discussion

This study investigated the relationship between long-term glycemic variability and neurocognitive performance in children with T1DM. Our findings demonstrated that higher HbA1c variability, expressed as both SD and CV, was significantly associated with lower full-scale IQ and poorer performance in executive domains, including working memory, processing speed, and executive function. Notably, HbA1c CV exhibited stronger correlations with cognitive decline than mean HbA1c, and it remained an independent predictor of neurocognitive impairment after adjusting for confounding variables such as age, duration of diabetes, hypoglycemia frequency, and socioeconomic status.

The present findings are consistent with previous literature suggesting that glycemic instability, rather than chronic hyperglycemia alone, plays a critical role in the development of neurocognitive deficits among pediatric T1DM patients [1-3]. Neuroimaging studies have consistently reported that glucose variability contributes to microstructural brain changes, including alterations in white matter integrity, which are associated with poorer cognitive performance [4,5]. These studies indicate that repeated exposure to fluctuating glucose levels may disrupt brain development and connectivity, particularly during critical neurodevelopmental stages. The stronger correlations observed in our study between HbA1c variability and cognitive outcomes further support this view, highlighting that cumulative glycemic fluctuations may have more profound neurotoxic effects than persistent hyperglycemia.

Our observation that working memory, processing speed, and executive function were most affected aligns with previous studies reporting that these cognitive domains are particularly vulnerable in T1DM [13,14]. Executive function deficits, encompassing cognitive flexibility, inhibition, and planning, have been widely documented in children and adolescents with T1DM and are often attributed to disrupted prefrontal subcortical circuitry caused by metabolic and vascular insults [2]. Moreover, studies have demonstrated a close association between white matter abnormalities on diffusion tensor imaging and lower processing speed scores, supporting a neuroanatomical basis for our findings [15,16].

In our cohort, children in the high-variability group also experienced a higher frequency of hypoglycemia. This finding corroborates earlier research suggesting that severe or recurrent hypoglycemia can contribute to cognitive dysfunction, particularly affecting attention and memory [17]. However, the association between hypoglycemia and cognitive decline has been inconsistent across studies, with some reports showing minimal or no significant long-term effects [18]. In our multivariate analysis, HbA1c CV remained a significant predictor of cognitive performance even after adjusting for hypoglycemia frequency, implying that glycemic variability itself may independently influence brain function.

DKA has also been recognized as an early risk factor for neurocognitive impairment in T1DM [19]. Although our study observed a non-significant trend toward more DKA episodes in the high-variability group, the lack of statistical significance could be attributed to the retrospective nature of data collection and the relatively small number of severe DKA events. Previous studies have demonstrated that children who experienced DKA at diagnosis exhibited structural brain changes and poorer cognitive outcomes years later [19]. Therefore, even though our results did not show a direct relationship, the role of DKA as a contributing factor cannot be ruled out.

The duration of diabetes and socioeconomic status were also found to have a modest but significant impact on cognitive outcomes. Longer disease duration has been associated with cumulative metabolic insults, leading to progressive microvascular and neuronal damage that may manifest as cognitive slowing [5]. Similarly, lower socioeconomic status may contribute to poorer cognitive performance through multiple indirect mechanisms such as limited access to healthcare, inconsistent glycemic monitoring, nutritional deficits, and educational disparities [6]. These factors likely interact synergistically with metabolic instability, exacerbating neurocognitive decline.

The mechanisms underlying the link between HbA1c variability and cognitive dysfunction are multifactorial. Glycemic fluctuations are known to induce oxidative stress, mitochondrial dysfunction, inflammatory cytokine release, and endothelial injury, all of which can compromise neuronal health and white matter integrity [2,3]. Experimental studies have shown that oscillating glucose levels lead to greater oxidative damage than sustained hyperglycemia, resulting in synaptic and myelin disruption [20]. Additionally, recurrent metabolic stress may impair cerebral autoregulation, contributing to subtle ischemic injury in developing neural tissues [21].

Our findings also support the concept of a “vicious cycle” between metabolic instability and cognitive dysfunction. Impaired executive function can compromise adherence to insulin therapy, diet control, and glucose monitoring, leading to greater glycemic variability. This, in turn, may exacerbate neurocognitive decline, forming a self-perpetuating loop that has been described in prior pediatric diabetes research [22].

The present study carries several important clinical implications. It underscores the need to minimize not only chronic hyperglycemia but also long-term glycemic variability as part of comprehensive diabetes management. Emerging technologies such as continuous glucose monitoring (CGM) and hybrid closed-loop insulin systems offer opportunities to improve TIR and reduce glucose excursions, which may ultimately mitigate neurocognitive risk [23,24]. Future research should focus on determining whether improved glycemic stability through such interventions can preserve neurodevelopmental outcomes in children with T1DM.

However, some limitations must be acknowledged. The retrospective design limits causal inference, and reliance on medical records may have led to incomplete data on hypoglycemia and DKA. HbA1c CV provides a reliable index of long-term variability but lacks the temporal granularity of CGM-derived measures. Furthermore, neurocognitive assessments were conducted at varying intervals, which could introduce temporal bias. Prospective longitudinal studies integrating CGM metrics, neuroimaging, and standardized cognitive batteries are warranted to validate these findings and explore underlying mechanisms in greater detail.

## Conclusions

In conclusion, our study demonstrates that higher HbA1c variability is independently associated with neurocognitive decline in children with T1DM, particularly affecting working memory, processing speed, and executive function. These findings emphasize the importance of maintaining stable glycemic control to protect brain development in pediatric diabetes. Addressing both mean HbA1c and its variability may be essential to preserving cognitive health and long-term quality of life in this vulnerable population.
